# Exploring Children’s Engagement in Monitoring Indoor Air Quality: Longitudinal Study

**DOI:** 10.2196/32404

**Published:** 2022-01-21

**Authors:** Sunyoung Kim, Gregory Sohanchyk

**Affiliations:** 1 School of Communication and Information Rutgers University New Brunswick, NJ United States

**Keywords:** children, indoor air quality, mobile app, awareness, longitudinal deployment

## Abstract

**Background:**

Indoor air pollution is harmful to everyone, but children are of particular concern, as they are more vulnerable to its adverse health effects from air pollutants. Although mobile technology is increasingly being designed to support monitoring and improving air quality indoors, little attention has been paid to its use by and for children. Previously, we created *inAirKids*, a child-friendly device to promote children’s engagement with monitoring indoor air quality through a participatory design process. The next step is to evaluate its usability in the real world.

**Objective:**

The aim of this study is to investigate how *inAirKids* affects children’s understanding of and engagement with indoor air quality through a longitudinal field deployment study.

**Methods:**

We deployed *inAirKids* in the homes of 9 children aged between 6 and 7 years, and investigated their use for up to 16 weeks by conducting semistructured, biweekly interviews.

**Results:**

The results show that participants promptly engaged with *inAirKids* but quickly lost interest in it owing to the lack of engaging factors to sustain engagement. In addition, we identified 2 design considerations that can foster sustained engagement of children with monitoring indoor air quality: design interactivity for engaging in continuity and corporate hands-on activities as part of indoor air quality monitoring for experiential learning.

**Conclusions:**

Our findings shed light on the potential to promote the engagement of children in indoor air quality as well as considerations for designing a child-friendly digital device. To the best of our knowledge, this is the first longitudinal field deployment to investigate how to engage children in monitoring indoor air quality.

## Introduction

### Background

It is widely known that air quality indoors, where people spend most of their time, is essential for occupant health and comfort [1]. However, it is not commonly known that indoor air in homes and buildings is typically more polluted than outdoor air, even in large and industrialized cities [2]. As many air pollutants are colorless and odorless, it is challenging to estimate air quality conditions using bare human sensors, such as eyesight or smell [3]. Thus, many people spend most of their time inside their homes without realizing poor air quality indoors and their association with health and well-being implications [4]

Owing to the advancement of personal and sensing technologies, smart devices are increasingly available in the market to monitor indoor air quality (IAQ). These devices have proven effective in improving IAQ, as simply making occupants aware of the IAQ levels in the homes can positively motivate their behaviors toward better IAQ [5]. However, most existing IAQ monitoring devices are optimized for interaction with adult users, which display air quality information using numeric figures, text, and graphs [6]. This trend leaves behind important household members who can highly influence and be influenced by IAQ, the children.

Not only do children spend the majority of their time indoors. They are also most susceptible to the effects of air pollution as their lungs are still developing, and they breathe in greater volumes of air per body mass than adults [7,8]. Furthermore, children can be highly influenced by education to influence improving IAQ in their household positively. Without relevant deliberations about users’ skills and cognitive abilities, the interface may cause confusion and even misunderstandings about the conveyed information [9]. As children’s needs, skills, and expectations differ drastically from those of adults, a technology designed for adult users may not be suitable for children to use [10]. Therefore, it is crucial to create a tool optimized for children to promote their engagement in monitoring and improving IAQ.

We previously investigated design considerations to best convey IAQ information to children in middle childhood (aged 6-8 years) through a participatory design approach [11]. In this previous study, we had children engage in the entire design process both as informants to express opinions on interacting with the device and testers to try out the prototypes and make suggestions for improvements. This process enabled us to capture the perspectives of the child, elicit a guiding principle of designing technology for children, and create child-friendly interfaces for IAQ visualization. On the basis of the findings from that study, this paper reports our field deployment study on *inAirKids*, an IAQ monitor that provides persuasive and expressive visualization of IAQ optimized for use by children.

### Objectives

Through a longitudinal deployment study of *inAirKids*, this study aims to investigate how the IAQ visualization designed for use by children affects the understanding of and engagement with IAQ by children and what contributes to or prevents the engagement of children in monitoring IAQ.

### inAirKids: App Design and IAQ Sensing

**Figure 1:**
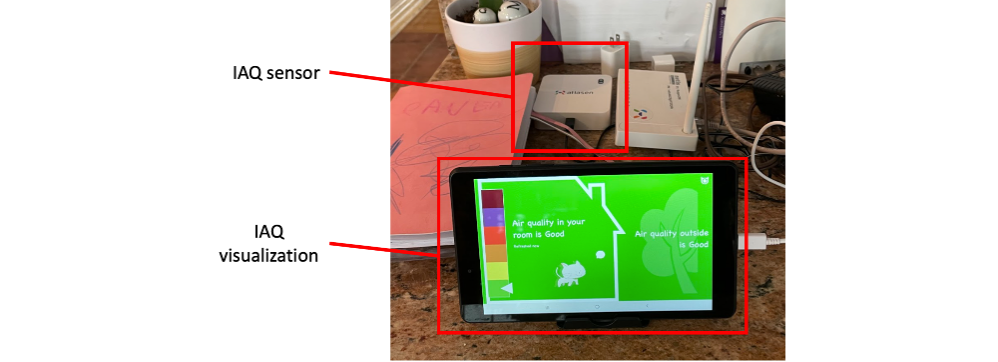
Setup of inAirKids. IAQ: indoor air quality.

**Figure 2:**
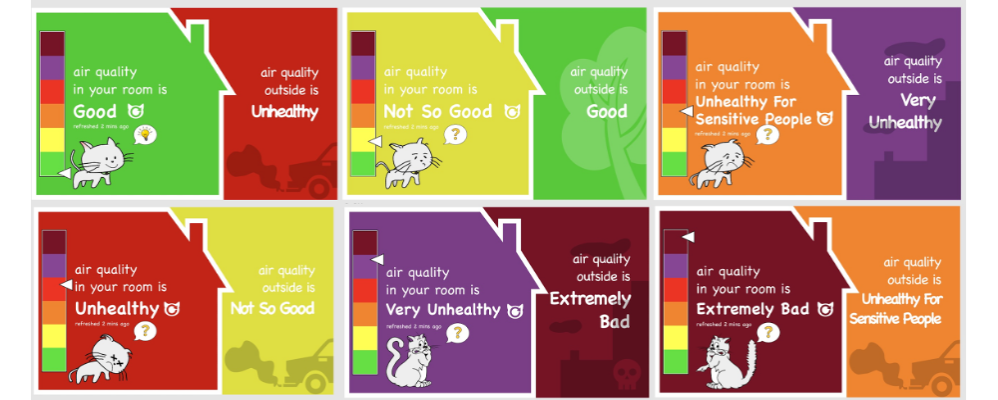
A set of indoor air quality visualization interfaces for inAirKids.

Our system, *inAirKids*, consists of a mobile app that runs on a tablet PC as a stationary device to represent air quality indoors and outdoors graphically and an IAQ sensor (Figure 1). On the basis of the findings from our previous study [11], we created *inAirKids*, a mobile app that runs on a tablet PC for children to check the current state of IAQ. In the design, we used a graphical metaphor of a house to visually illustrate air quality indoors and outdoors in a child-friendly manner (Figure 2).

**Figure 3:**
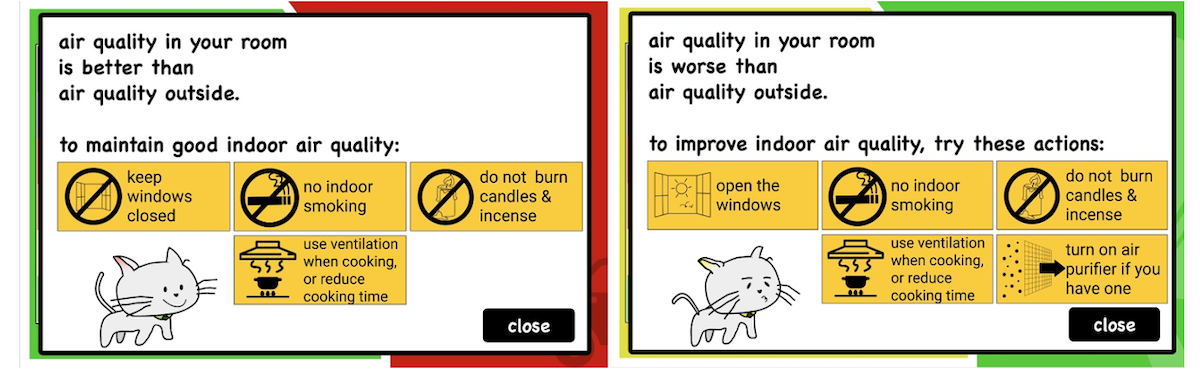
A popup screen of inAirKids that describes the current indoor air quality status with interventions.

In designing *inAirKids*, we used various graphical elements to meet the abilities, skills, and perspectives of children. First, we used simple language to explain the level of air quality so that young children who can read can easily comprehend it. The six labels to indicate different air quality states by the air quality index (AQI) of the environmental protection agency are good, moderate, unhealthy for sensitive groups, unhealthy, very unhealthy, and hazardous [12]. As some of these labels are not easy for children to understand, such as *moderate* and *hazardous*, we changed them to *not so good* and *extremely bad*, respectively. Second, we applied 6 colors from the AQI color codes to inside and outside a house graphic that directly compares the current air quality indoors and outdoors. Third, we added an animating cat that strolls inside the house to respond to different IAQs and narrate its meaning. For instance, a cat smiles and moves lively around the house when the IAQ is good, but it frowns and moves sluggishly when the IAQ is poor. Fourth, we applied relevant background images outdoors to portray air quality outside (eg, tree for good, automobile exhaust for not good, and factory chimneys for bad). Finally, clicking a cat will display a popup screen that describes the current IAQ status and suggests proper actionable interventions for children to improve the IAQ (Figure 3).

**Figure 4:**
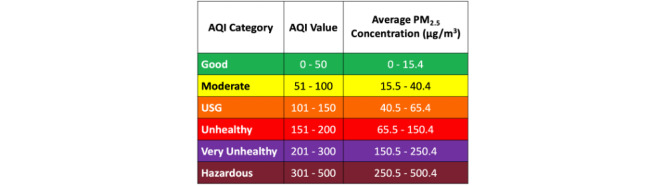
The air quality index category for PM2.5 (from AirNow [13]). AQI: air quality index; PM: particulate matter; USG: unhealthy for sensitive groups.

For IAQ sensing, we used an off-the-shelf sensor that continuously measures the levels of five indoor air pollutants: fine particulate matter (PM_2.5_), carbon monoxide, carbon dioxide, total volatile organic compounds, and nitrogen dioxide (Figure 1). This sensor transmits the measurements of these air pollutants to the server every 15 seconds. The server then sends the current IAQ level to the app every 5 minutes. The system determines the current IAQ level based on the level of air pollutants that AQI falls under as the lowest category among the 5 air pollutants. For instance, if the 5-minute average of PM_2.5_ is 20 μg/m^3^ and its AQI category is the lowest among the air pollutants, *inAirKids* displays IAQ as *not so good* (Figure 4 [13]).

## Methods

### Participant Recruitment

Children aged 6-8 years and who could read were eligible to participate in the study for up to 16 weeks. We chose the age range of 6-8 years, as children in this age group begin to read and use digital devices with information written in simple languages for learning and reasoning [14]. After obtaining approval from the Rutgers institutional review board, we distributed recruitment fliers on social media and local community groups on the web for parents on Facebook, NextDoor, Reddit, Twitter, and others. The recruitment flier included the study purpose, duration, participation criteria (children aged 6-8 years who can read), what children are asked to do, and monetary compensation. After confirming a child’s age and readability, we obtained temporary consent from parents about the participation of their child in the study by phone. Parents and children provided written consent for participation during the visit of a researcher to their home for the device setup. In all, 11 children were recruited to participate in the study, 4 (36%) female participants and 7 (64%) male participants, (mean age 6.5 years, SD 0.7 years; Table 1), 4 (36%) participants of whom had withdrawn within the first few weeks of the study.

**Table 1:** Participant demographics and study duration.

ID	Age (years)	Gender	Study completion	Study duration (weeks)	Number of interviews
1	8	Female	Completed	16	8
2	7	Female	Completed	16	8
3	6	Female	Completed	12	6
4	7	Male	Completed	12	6
5	7	Male	Completed	12	6
6	6	Male	Completed	12	6
7	7	Male	Completed	12	6
8	6	Male	Withdrawn	6	3
9	6	Female	Withdrawn	6	3
10	6	Male	Withdrawn	2	1
11	6	Male	Withdrawn	2	1

### Consent and Withdrawal

#### Overview

Although we obtained consent from all participants before the study, we considered consent as an ongoing process to renegotiate verbally throughout the study duration. As children are often less familiar with what research entails, they may initially wish to participate but later feel less keen as they realize what is involved in the study [15]. Alternatively, parental consent obtained as a safeguard to protect children may restrict the ability of children to participate voluntarily in research [16]. Considering all these, children need to feel comfortable ending their involvement in the research should they wish to do so from an ethical standpoint.

Among the 11 children who signed up for the study, 4 (36%) children, all aged 6 years, had withdrawn from the study within the first few weeks because they did not have or lost their interest in the study. For those who answered “I do not know” to most of our questions in the interview, we explained to feel comfortable to end their involvement in the study whenever they wanted. In all, 2 (18%) participants expressed their willingness to withdraw from the study after 1 interview, and 2 (18%) other participants did so after 3 interviews. After explaining to a parent about the right of their child to participate or withdraw voluntarily in research, we removed them from the study. We discarded all data collected from 2 (18%) children who had withdrawn after the first interview. We kept the data from the remaining participants for data analysis, which made 9 (82%) participants in total.

#### Data Collection

We conducted biweekly interviews with the participants to collect qualitative data on the use of *inAirKids* by children over time. All data were collected through interviews using a videoconferencing software of the choice of the participant (eg, Skype or Zoom). In addition, we made 2 visits to the home of the participants for device setup before launching the study and its pickup after the study was complete.

#### Interview Protocol

Our interview focused on the following four aspects: (1) how children initially perceive and respond to *inAirKids*, (2) how they use it in their daily lives, (3) what motivates or prevents their use of the device, and (4) how their engagement in IAQ changes over time. On the basis of this, we constructed a set of open-ended interview questions in 3 phases of the study duration to explore these spaces. The first phase focused on understanding the purpose of participating in the study, general perspectives about IAQ, and initial impressions of *inAirKids* in the first interview. The second phase focused on exploring the user experience in-depth, including patterns of using *inAirKids*, engagement in IAQ, and factors contributing to or preventing engagement of children in monitoring IAQ throughout the deployment duration, except for the final interview. Finally, the third phase focused on exploring suggestions for system improvements and reviewing the overall reflection on the use of *inAirKids* in the final interview.

#### Study Procedure

**Figure 5:**
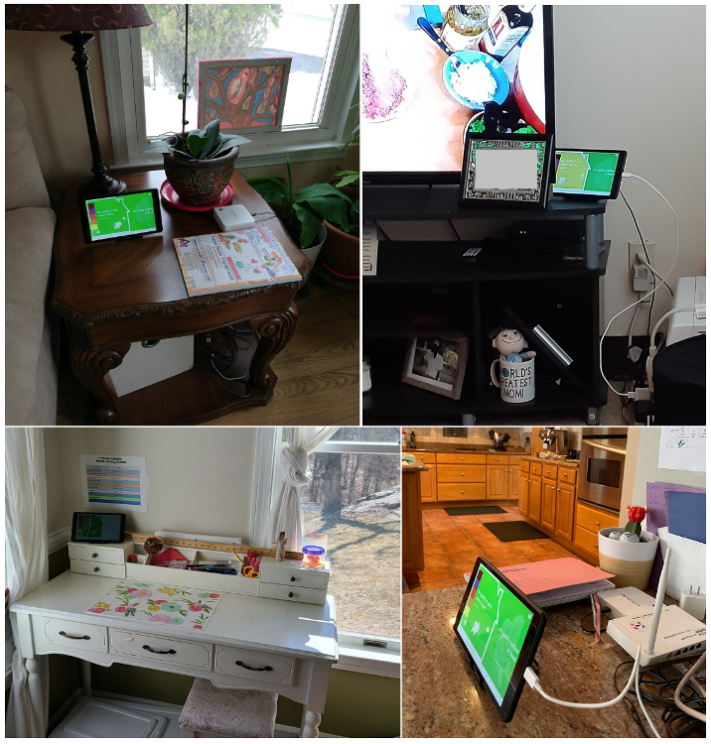
The location of a tablet PC includes a side table or a television stand in a living room, on a dining table in a kitchen, and on a desk in a child’s study.

Before the study started, the research team visited the home of a participant to set up *inAirKids* in the location of their preference (eg, a nightstand or a coffee table). Participants and their parents were asked to place the *inAirKids* display (a tablet PC) anywhere in the house to see it easily in their everyday lives (eg, a living room, a dining room, or a study; Figure 5). After setting up, we introduced *inAirKids* to the participants as “a device to present air quality both inside and outside of the house in real-time.” We then provided basic instructions on how to use the app. In addition, the parents of the participants filled out a survey to inform us about their basic demographic information, including the age of the child, household type, income, purpose of participation, and ethnicity. Finally, both participants and their parents were told to freely interact with *inAirKids* as much as they wanted throughout the study period. In addition, they were given contact information from the research team if they needed technical support.

During the interview, participants were told to freely use any materials (eg, pen and paper) to supplement their feelings or thoughts to facilitate their engagement in the study [17]. When they drew on a paper, we asked to show them on a screen to take a screenshot or a parent pictured and emailed them to us. When a child expressed no interest in answering questions or when a researcher had difficulty engaging them in the interview, we asked how much they liked participating in the study. We explained that they could withdraw from the study at any time and asked if they wished to terminate an interview early. Although all the interview questions were for children, we allowed the parents of the participants to join the interview and share their thoughts and opinions when they wanted. Most parents (mothers) participated in the interviews.

After the study was complete, the research team visited the homes of the participants and picked up the device. All participants were compensated with a gift card for their time up to US $160, prorated by the duration of participation after device pickup. Participating in this study did not have any harmful consequences on the health of the participating children.

#### Study Duration

The duration of deployment was initially planned to be 16 weeks. However, we shortened it to 12 weeks in the middle of the study, as many participants lost interest in the device as they continued using it gradually, but less significantly. Thus, 18% (2/11) of the participants completed the study for the entire 16 weeks, and 45% (5/11) of the participants completed the study for 12 weeks. We conducted interviews with each participant every other week for the duration of the study. Thus, we conducted 8 interviews with 18% (2/11) of the participants who participated in the study for 16 weeks and 6 interviews with 36% (4/11) of the participants who participated in the study for 12 weeks. Each interview lasted between 30 minutes and 1 hour. All interviews were audio recorded and transcribed.

#### Data Analysis

We analyzed the interview data using thematic analysis to reveal patterns across data sets and find significant themes through open, axial, and selective coding [18]. First, we conducted open coding to identify and code concepts significant in the data as abstract representations of events, objects, happenings, actions, and so on. The example excerpt below illustrates how one participant lost interest in using the device because of no change in the app interface. This response is coded as *bored_of_continuity*:

{bored_of_continuity}I didn’t look at the tablet at all this past week at all because nothing’s changing. It’s been green all the time. It’s boring.{/bored_of_continuity}

P2

Next, we categorized the related concepts created by open coding into conceptual phenomena using axial coding. Phenomena refer to repeated patterns of events, happenings, actions, and interactions that represent people’s responses to problems and situations. For instance, *losing_interest* refers to a participant’s loss of interest in using our app and associated factors that contribute to it. During axial coding, the open code *bored_of_continuity* in the example excerpt above was categorized as *losing_interest*, as it illustrated how the participant began to lose interest in using our app. Finally, we followed the selective coding process to assemble the conceptual phenomena extracted from the axial coding. The goal of this step is to integrate all concepts by building relationships across phenomena.

## Results

### Early Phase of the Study: Positive Initial Interaction With inAirKids

Overall, we received positive feedback about the design of *inAirKids* after their initial interactions with it, as it was easy to understand the current IAQ status from its display*.* Except for those who withdrew, most participants engaged swiftly with *inAirKids* immediately after installation. Without much instruction, they quickly figured out how to interpret various visualization components on *inAirKids* and became aware of how different indoor activities affected IAQ differently:

It’s very easy to use. I think any kid can easily understand it. The colors are easy to understand. It’s like you are good to cross the road when the traffic light turns green.

P1

The color coding is nice. The instructions are very clear, so you don’t really need to you know fully understand English or even science to kind of utilize it, so that’s good. That’s the best part about it.

P5

I check it every time I come by it, like when I wake up in the morning or when I go to sleep at night. I basically do it every day. After school, before I eat breakfast, before I eat lunch, before I eat dinner. It’s important to know how good or bad the air quality is so that I open the windows when it’s bad.

P2

When we asked the participants to describe their experiences of using *inAirKids* during the early phase of the study, many dialogues were made from or reflected from the perspective of the animated cat on *inAirKids*. We implemented several visual components to illustrate the different levels of IAQ within *inAirKids*, including an animated cat, a silhouetted house, and AQI-indexed colors. Among these components, the animated cat that responds to different IAQ levels was found to be effective in drawing the participants’ attention to and helping them engage in monitoring IAQ. We found that the cat served as a proxy for the participants to experience and respond to different IAQ levels indirectly, making their interaction with *inAirKids* as personified experiences, which echoes a previous work [19]:

The first thing I notice is a cat walking to the side of the house. Every time I walk by, I see the cat. The cat has Xs on his eyes because he is sick when the air quality is not good. I like the cat because it tells us what to do and like it shows the emotions.

P3

The cat is happy when the air is good. If the air wasn’t as good, the cat would be sad. When it [the tablet] is red, he [the cat] walks very slow. Then when it starts to turn purple, his tail starts sticking out, his hair is down, and then his tail’s wiggly, and his hair goes like that [spikey]. He gets scared when the air quality is worse. Then, we opened some windows, so the cat is not sick. Make it fresh air in the house. Then the cat doesn’t feel sick anymore.

P4

The cat is mad when we are cooking. He wants the air quality to be good every day. Mad kitty, he has to understand that the air quality can be bad sometimes, like when we are cooking bacon.

P7

Within the first few weeks, the participants swiftly noticed that cooking and opening windows are the 2 activities that significantly influence IAQ negatively and positively, respectively [20]. When these activities occurred, they fully engaged with *inAirKids* and monitored IAQ until the color of the display changed back to green—good IAQ:

It [inAirKids] is green now. It was yellow probably an hour ago when the Airwick is on. It changed to green now because I opened the window.

P2

When my mom was cooking, it becomes yellow. I was surprised because it stayed in the yellow for like five hours. We opened some windows so it can’t get bad.

P3

It [inAirKids] does not like the bacon smoke. I would probably say it hates it. It turned to red when my mom was frying. So, most of the time, I look at it when my mom is cooking. And I keep the kitchen door open until she’s done cooking.

P6

### Getting Used to Using inAirKids: Prolonged Engagement in IAQ

As the study proceeded for a few weeks, we found that the participants started to develop their ways for prolonged engagement with IAQ. It was implemented primarily in two ways: soliciting the involvement of parents in improving IAQ and converting IAQ monitoring practices to art and craft activities.

### Soliciting Involvement of Parents for IAQ Improvement

One of our assumptions of this study was that, if properly informed and educated about IAQ, children can positively influence their family members to become involved in improving IAQ. Our findings confirmed this assumption. Regardless of the location of *inAirKids*, we observed that the participants were the primary users of the device and other household members, especially parents, were prompted to be involved in IAQ monitoring by the child participant.

Our findings show that the increased awareness of IAQ among children promoted their parents’ inclusion and engagement in improving IAQ. When they noticed IAQ worsening, the participants actively sought the involvement of their parents in improving IAQ by telling them the IAQ status and asking them to take proper actions to reduce air pollutants. Most parents shared their experiences of their child asking, and sometimes even pestering, they took prompt action when their IAQ was not *green*. In fact, the involvement of parents was essential for IAQ improvement, as a child cannot execute IAQ interventions, such as unlocking a window to open or controlling heating, ventilation, and air conditioning appliances:

It gives me a good sense of protection. I can tell my family when to open the windows, when to close the windows...The other day, I saw that mommy had a candle lit, so I told mommy to blow it out. It has dirt and dust in the smoke and makes it bad. I tell her that candles are bad for the air because the smoke from the candles makes the air bad.

P2

He would always notice what the air quality was like and then come and tell me. Then, I would have to turn on the vent or the window. Especially when we were having construction for a couple of weeks, it was constantly turning yellow and orange. So, he was very much wanting to make sure that we were doing something about that.

Mother of P4

He was trying to open the window when he saw the tablet was orange. He asked me to help him open the windows because they have the two special locks on them, so it’s hard for him to open the windows.

Mother of P6

### IAQ Monitoring as Art and Craft Activities

**Figure 6:**
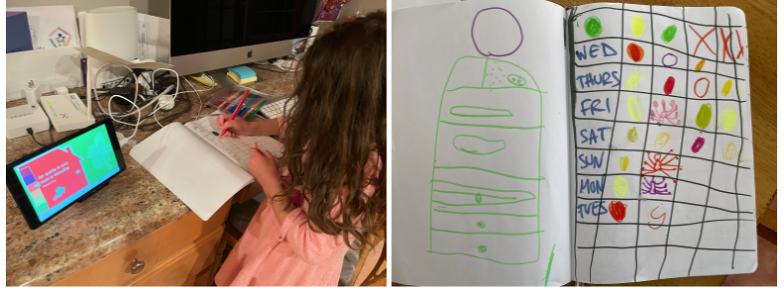
Journaling a daily indoor air quality status (left) and a journal (right).

Although we asked the participants nothing but to interact with *inAirKids* for the study, many participants expanded their interaction with *inAirKids* to various hands-on art and craft activities as part of their IAQ monitoring practices. For instance, 1 participant created a journal of IAQ after using *inAirKids* for about a month. She then kept a daily record of IAQ in which she drew color-coded bugs and icons to mark different IAQ levels (Figure 6). This journaling activity had quickly become part of her daily routine, and she continued doing it for the rest of the study. This gave her extra motivation to monitor IAQ regularly, not as a passive recipient but as an active author, cocreator, and inquirer of information, which a previous work referred to as *active learning* [21]:

I keep the journal to keep track of air quality every day. It’s fine when it’s green or orange, and I mark a circle. But when the tablet is red or purple, it’s not good, and I draw a red spider or a purple spider.

P2

**Figure 7:**
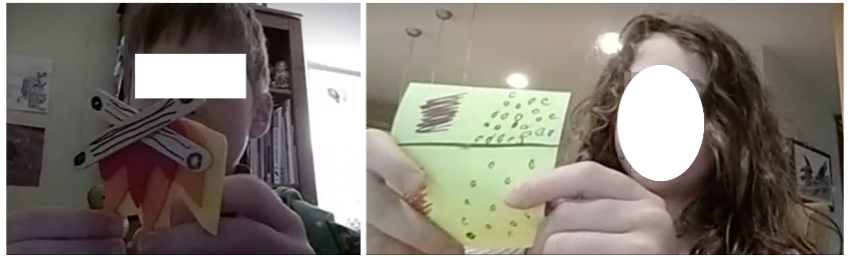
Art and craft activities for monitoring indoor air quality, including crafting a bonfire to depict a source of air pollution (left) and drawing air pollutant particles in different densities to illustrate different indoor air quality levels (right).

In addition, the participants created various drawings and crafts throughout the study period as part of their IAQ monitoring practices. It included a crafted bonfire to illustrate a source of air pollution or a drawing of air pollutants at different densities to depict different IAQ levels (Figure 7). Some of these activities were initially suggested by parents. However, most participants mentioned that they engaged in and had fun in the art and craft activities as part of IAQ monitoring.

In addition, many parents expressed satisfaction with their child’s learning and engaging with IAQ, which was the primary purpose of participating in this study, the desire of a parent for their child to learn about IAQ from an educational standpoint. Furthermore, these activities led to conversations and discussions about air quality between parents and children, which positively influenced and further promoted inquiries by children about air quality in general:

We talk about stuff like air quality in the house a lot. So, it’s given us another topic to talk about. She learns about stuff in school with the environment and being environmentally conscious. And this [inAirKids] adds another layer to that for her. We were having discussions that we would not have had otherwise, like what would make air quality good or bad. It gave me an opportunity to have a teachable moment with her for her to learn more about air quality and environment.

Mother of P2

He asks about air quality when he goes to different places, like his grandma’s house. He also asked what air quality would be like when he farts or poops. And the other day, he moved the air quality sensor to the bathroom.

Mother of P5

I think it’s made her aware of the terms. At six years old, it’s not something that we would really be having conversations about. Now she’s familiar with the term air quality, and it’s been something that’s ingrained in her head.

Mother of P3

### Attenuated Interest in inAirKids: Disengagement From IAQ

As the study proceeded for several weeks, the participants exhibited a strong novelty effect on *inAirKids*. Unlike the first few weeks when the participants fully engaged with *inAirKids* for IAQ monitoring, we noticed a significant decrease in their engagement after several weeks of the study. Two key factors attributed to this phenomenon include participants’ learning of IAQ change patterns over time and our focus on designing *inAirKids* to deliver IAQ changes.

### Learning the Patterns of IAQ Changes

The participants gradually learned the patterns of IAQ changes as the study proceeded. *inAirKids* enabled the participants to quickly determine two indoor activities that act as the primary source of indoor air pollution: cooking and burning candles. Once they recognized this, the onset of these activities, not *inAirKids*, triggered participants’ interest in and attention to IAQ. The positive aspect is that it demonstrates the effectiveness of *inAirKids* in teaching children about primary sources of indoor air pollution. Meanwhile, it echoes a previous study about the rapid loss of interest of a child in a toy [22], showing that *inAirKids* was not successful in addressing it:

Before she was like checking it in the morning, before breakfast, go to school, come home, get off the bus, go look at it after dinner and before bed. Now she knows that if there’s no cooking or candles or anything, the air quality is probably fine, and there’s no reason to check.

Mother of P3

He was attentive to the air quality and asked like opening the window, turning on the fan, and telling me to do that stuff like the first three or four weeks. Then, it’s tapered off since then. He knows what kind of things we would be doing that would make the air quality bad. So, if there are no candles or if I’m not cooking something weird, he assumes that it’s going to be green which has kind of been the case.

Mother of P5

In addition, the participants gradually got used to having moderate levels of IAQ. During the first few weeks, *inAirKids* displaying any color other than green prompted the participants to take immediate actions to make the color green. However, as the study continued, they became accustomed to and concerned less about the slight worsening of IAQ, such as *inAirKids’* color being yellow or orange. It is known that IAQ changes constantly and that people get used to recurrent upheavals. Thus, it is not surprising that the participants developed relaxing attitudes toward moderate levels of IAQ as the study proceeded. Meanwhile, it demonstrates that *inAirKids* was not successful in retaining the attention of the participants to the recurrent worsening of IAQ, which is crucial to enhance the overall IAQ [5]. As they learned that IAQ could worsen somehow, the color change in *inAirKids* from green to yellow or orange was not an event to pay attention to anymore:

It just didn’t have as much of a focus. It’s always either green or yellow. I think she’s in a way found comfort as she’s never seen it get worse either. Originally, she was like pretty hung up the first couple of times when she saw it yellow. And as she realized that sometimes it is yellow and it’s not terrible, but it’s okay, she didn’t seem as concerned about it because I think it just became more normal to her.

Mother of P6

In the beginning when it first would change, he would notice that it was yellow or orange, and he would be concerned, liked to cover his face, and asked questions like what’s happened and why it is like that. And I explained to him why it’s yellow and stuff. Now he’s a little bit easy about it unless it would go up to red or something. Today, he might still be concerned, but if I just light a candle and it’s yellow for ten minutes, it’s not a big deal.

Mother of P7

### inAirKids Designed to Deliver IAQ Changes

In designing *inAirKids*, we focused on displaying the current state of IAQ in ways in which a child can easily understand and act on different IAQ statuses appropriately. To that end, we created *inAirKids* with background color changes corresponding to the current IAQ status. Then, it turned out that the IAQ of most participants’ houses was mostly good. Thus, the background color of *inAirKids* remained green most of the time for most participants, except when indoor activities such as cooking or burning candles occurred. Consequently, one of the most prevalent comments we received during the later phase of the study was *inAirKids* being *boring*:

I checked the tablet every day right after I’m done with my work, I come in and check on it. Now I have not looked at the tablet at all because I get bored of it. It’s boring because it never changes color. The color is green all the time.

P2

When the color goes up, it’s interesting to see it because you can see the different colors. But it isn’t as interesting because the colors never go up or down. I want to see if the tablet can get to different colors. But it never turns any color but green. When it goes to different colors, I get more excited because when it’s about the same color for like a long time, it gets kind of boring.

P4

We intended to encourage the engagement of children in monitoring IAQ for its improvement, which took place during the early phase of the study. However, the attention of the participants moved to capturing the moment of color change in *inAirKids,* as the study proceeded. They perceived the moment of changing colors in *inAirKids* as an exciting event to capture, whether it was improving or worsening IAQ. When explaining their experience with *inAirKids* in the interviews, they were excited to share the moments they noticed color changes in *inAirKids* regardless of whether IAQ improved or worsened. In contrast, they disappointedly shared their experiences when they did not see any color changes, even though their IAQ remained good all the time. As it was designed to highlight IAQ changes, our participants, young children, felt bored when IAQ stayed good, as the color of *inAirKids* did not change. Some participants even tried to make the color change by relocating its IAQ sensor:

I wanted to see what the air quality in the bathroom was like because we’ve never put it in the bathroom, and I was happy to see the color change. Because it’s not so exciting when it just keeps one color for a lot of the time, and it’s cool to see the color change to a different color than green. I want to make sure that everyone’s healthy in my house, but also, it’s exciting to see the color change.

P4

My grandma was frying, and I moved the sensor closer to the kitchen to see if the air quality would turn a different color because it would be interesting to see it turn to a different color.

P7

### Suggestions for App Improvement

**Figure 8:**
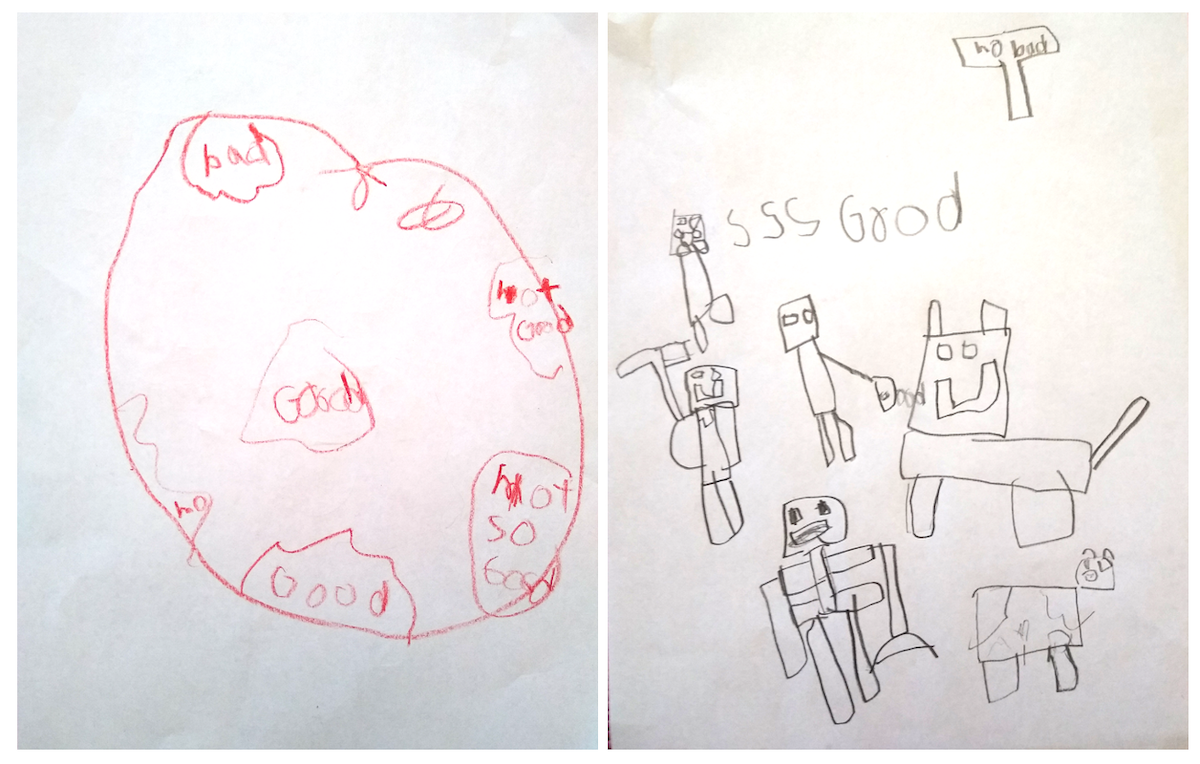
Drawings of Earth with different levels of air qualities in different regions (left) and various Minecraft characters that respond to varying levels of air qualities differently (right).

In the final interview, the participants were asked to share their thoughts on improving *inAirKids* to better meet their needs. Some participants had already explored their versions of *inAirKids* as part of their hands-on arts and craft activities (Figure 8). The most prevalent response to our interview question, “What changes would you make to *inAirKids* if you would remake it?” was to add more diversity and interactivity. This aligns with the most prevalent complaint of *inAirKids* being boring.

The participants suggested adding more features to the background for diversity. We thought that an animating cat on vivid background colors would be simple yet effective in delivering IAQ information to children. However, as their interaction with *inAirKids* continued, our participants found it too simple and sought more variety in the interface. Probably because *inAirKids* illustrated a loitering cat inside a house, the participants suggested adding various other animals in various circumstances, such as a panda in the wild or a penguin and a bear in Antarctica:

I would love to add more animations and different characters. I’d add the National Geographic background with random animals, like pandas, llamas, bears, lions, jellyfish. Or, it would be fun if there would be a way to change your background to a different setup in order to be on the moon, which you have to unlock it.

P5

I want more changes in the background. I would add a flying penguin to the Antarctica background. Or, I would add a happy polar bear and an arctic hare which will follow the kid everywhere he goes in the background.

P6

In addition, the participants suggested implementing more interactivity to the app, especially when IAQ changes occurred. Many such suggestions were much more drastic than a simple background color change that we implemented in *inAirKids*, from wearing a mask to erupting a volcano:

I think adding more characters like dogs or dinosaurs would make it interesting. The dog would go sniffing around and bark when the air quality gets bad. Or the dinosaur would be wearing a gas mask when the air quality would be bad so that it can help you understand it more.

P1

I really want there to be like something strange or rare happens depending on how bad the air quality is. Like, the first is when the air quality is good, the cat gets elected president. And when the air quality is bad, a volcano erupts.

P5

## Discussion

On the basis of our findings, we discuss the considerations and lessons learned to design digital tools that would help children monitor and improve IAQ. Although our discussion centered on a mobile app for IAQ monitoring by children, we believe these considerations can be applicable to creating digital tools for educational or scientific inquiries for children in general.

### Difference Between Results in a Participatory Design and a Field Deployment

We designed the *inAirKids* interface through a robust and iterative participatory design process. The participatory design approach is a design process in which potential users, partnering with designers, are actively and directly involved in designing end user products [23]. In general, the participatory process involves brainstorming and low-tech prototyping tools to capture and demonstrate the ideas of the participants [23]. In this approach, the participants provide user-centered insight into the design, explain their difficulties with existing materials, and evaluate the interactivity [24]. Children aged between 7 and 10 years are considered ideal for participatory design because of their *emerging capacity for reflection and abstraction, and their lack of preconceptions about the design domain* [25]. Thus, the participatory design approach has been extensively used to design technology for children [26-28] and has become one of the most prevalent usability methods in the fields of human-computer interaction for children.

All design decisions for *inAirKids* were made based on careful consideration of the child participants’ feedback and comments from the participatory design. When we tested the working prototype of *inAirKids* with children, most of the feedback was positive and promising. In addition, we sought to address all issues in the interface design and usability brought up during participatory design in its development. However, we evidenced a clear sign of a novel effect as our longitudinal deployment study proceeded. Although initial feedback during the early phase of the deployment was positive, similar to those from a participatory design, responses of the participants vastly changed as their use continued. What participants praised as advantageous in participatory design disappeared quickly. Instead, many participants pointed out the shortcomings of the *inAirKids* interface as they had become used to it. Most complaints were related to the lack of interactivity and diversity of the app, which can only be captured through the extended use of a device via longitudinal field deployment.

This result provides empirical evidence regarding the real-world effects of different methodological approaches—participatory design and field deployment—in designing a digital tool. Although the consequences of the participatory design approach are invaluable to ensure the usability of a system from a user-centered perspective [25], some aspects such as a novelty effect cannot be captured from short-term user interaction. Thus, researchers and practitioners should not solely rely on study results but should also critically evaluate how the outcomes of different methodological procedures might unfold as a technology is used in the real world.

### Incorporating Hands-on Activities in IAQ Monitoring for Experiential Learning

Research has shown that playful and investigative activities support the engagement of children [29], which our findings echo. Although we did not ask, most participants voluntarily engaged in various hands-on art and craft activities as part of their interaction with *inAirKids*, making their practice of IAQ monitoring more fun and enjoyable. We deem this practice of *experiential learning*, in which children expand their engagement with *inAirKids* from passively receiving information to actively interpreting information through concrete hands-on experiences.

Experiential learning is a specific type of learning that connects the experiences of children to learning objects [30], thus enhancing the adaptation of new skills and knowledge [31]. Experiential learning has been shown to help students improve their understanding of scientific concepts and promote their learning interest by abstracting conceptualization from concrete actions [32,33]. Thus, children can enhance their comprehension based on their embodied experiences to construct conceptions and relationships actively while engaging in hands-on activities [34].

When creating *inAirKids*, we did not consider incorporating any hands-on activities into its use. Most existing IAQ monitoring devices have a user interface that displays air quality information using numeric figures, text, and graphs [6]. Thus, we focused on creating a child-friendly user interface with versatile graphical components and animated characters. This left behind exploring the opportunity to facilitate hands-on activities, a practice that children can engage in for a better learning experience and embodied cognition [35]. We overlooked this aspect, perhaps because we are accustomed to creating a digital tool for adults primarily to provide information. User experience with *inAirKids* might have been more positive and engaging if relevant hands-on activities were systematically implemented as part of the *inAirKids* system. Examples of such activities include providing suggestions for various art and craft activities relating to current IAQ levels, applying drawings of children to the interface to personalize an animated cat, printing out a coloring book for different IAQ levels, offering an electronic drawing board feature as part of an interface, and many more.

As toys are increasingly digitized and screen-based, it is worthwhile for researchers and practitioners to explore ways to enable children to interact simultaneously with both digital information and the physical world. Studies have demonstrated the suitability of digital technologies, particularly mobile technologies, in facilitating experiential learning opportunities for children [33,34,36]. Our findings emphasize the importance of considering this approach for better engagement and learning by children in designing a digital tool for children’s scientific and environmental inquires and beyond.

### Designing Interactivity for Engagement in Continuity

In general, the interface design of a personal monitoring device focuses on capturing and delivering the event of something special happening. For instance, the Fitbit vibrates if the user reaches the daily personal goal, a smoke detector beeps to alert gas leakage, and a home security system notifies when a door or window is opened unexpectedly. It is appropriate to design these devices to capture and deliver the changes, as they need to draw the attention of the users when something special happens. When designing *inAirKids,* we followed this practice by focusing on attracting the attention of a user when IAQ changes occur. To that end, we made the background color of *inAirKids* change when the IAQ level changed as its primary interactivity component.

Our findings showed that the background color change effect was played as intended. It effectively drew the attention of a user to *inAirKids* and helped children quickly apprehend their current IAQ status. However, the issue was with the frequency of its occurrence. Fortunately, the IAQ levels of most participants’ houses were good most of the time, except when certain indoor activities, such as cooking, were happening. Consequently, background color changes seldom occurred. A good IAQ means that IAQ poses little or no potential to affect health, and thus, it should be perceived as favorable. In contrast, some participants felt disappointing and bored to maintain good IAQ, as they perceived the color change effect, which rarely occurs, as fun, entertaining, and exciting.

We received many requests to add more visually stimulating and animated effects to the *inAirKids* interface during the third phase of the interviews. Although these are all invaluable feedback, how to apply these requests needs to be critically reviewed. Although this can be implemented by simply adding more features when delivering something happening (eg, IAQ changing), an equally, if not more, effective approach would be to make the stable condition (eg, IAQ being good for a while) engaging and fun. We believe that the experiences of the participants with *inAirKids* were not as rich as we hoped, as our focus was on designing interactivity only for changes, which prompted the participants to engage in IAQ changes. Although it is worth further discussing whether children need to be attentive to IAQ when it is always good, it is crucial to deliberate how a different design focus can shape the user experience differently. Depending on the context of use, target users, and the purpose of a digital tool, different design foci for interactivity must be explored, including delivering changes, rendering continuity, or both.

### Limitations

Our findings must be evaluated in the context of several limitations. First, our sample size was small, and the attrition rate was high among younger children. Thus, our participant pool may not be representative of the general population of children. Second, the study duration was different among different participants, which runs the risk of compromising validity. As a novelty effect is strongly related to the duration of use [37], we might have captured fewer incidences of the findings relating to a novelty effect from those who completed the study earlier. We then collected strong evidence of a novelty effect even from those who completed the study earlier, demonstrating that the novelty effect persisted earlier in the study. Third, the overall IAQ was good mostly in all participants’ houses, which must have influenced how they interacted with *inAirKids* and perceived IAQ in general. If children living in more polluted areas were recruited, the results might have been different. Although we believe our findings provide valuable insights into understanding how children living in healthy indoor environments would interact with *inAirKids*, a further study is needed to investigate how children in a different sociotechnical status (eg, low-income families) or living in air-polluted regions (eg, near factories, high-traffic areas, and low-income countries) would interact with *inAirKids* and how their experience would influence engagement in IAQ differently.

### Conclusions

As time spent indoors increases in modern society, the impact of indoor environmental quality on comfort, health, and productivity of occupants also increases. With the advancement of personal computing and sensing technologies, there has been an increased interest in using sensors and smart devices to promote the engagement of occupants in monitoring and improving IAQ. However, most existing IAQ monitoring devices are optimized for interaction with adult users, leaving behind important household members who can highly influence and be influenced by IAQ, the children. We investigated how *inAirKids* affects the children’s understanding of and engagement with IAQ through a longitudinal deployment study. Our findings shed light on the potential to promote the engagement of children in IAQ as well as considerations for designing a child-friendly digital device. To our knowledge, this is the first longitudinal deployment study to investigate how children engage in IAQ monitoring. We hope that our findings will encourage future studies on the engagement of children with indoor environmental quality.

## Abbreviations

AQI: air quality index
IAQ: indoor air quality
PM: particulate matter
